# How the Cambridge Crystallographic Data Centre Obtains its Information

**DOI:** 10.6028/jres.101.038

**Published:** 1996

**Authors:** David G. Watson

**Affiliations:** Cambridge Crystallographic Data Centre, 12 Union Road, Cambridge CB2 1EZ, England

**Keywords:** CIF format, electronic deposition form, private communication

## Abstract

This paper is concerned with the acquisition of supplementary information, both hard-copy and electronic. Special arrangements with major journals are discussed and plans announced for the deposition of private communications using an electronic deposition form.

## 1. Introduction

Until very recently, the Cambridge Crystallographic Data Centre (CCDC) has obtained essentially all of its information from the scientific journal literature. Whereas Acta Crystallographica used to be the predominant journal for the publication of crystal structures, the situation is now very different as shown by the statistics covering the years 1990–1994.

**Table:** 

Source	1990	1991	1992	1993	1994
American Chem. Soc.	28 %	28 %	29 %	30 %	30 %
Germany, Switzerland	10 %	12 %	12 %	14 %	15 %
Royal Soc. Chemistry	13 %	12 %	13 %	13 %	13 %
Acta Crystallographica	13 %	13 %	11 %	10 %	11 %
Other Journals	36 %	35 %	35 %	33 %	31 %

## 2. Data Validation

Irrespective of the source, all data are validated by well-tested check software. The crystal connectivity is generated, reported bond lengths are compared against recalculated values and the crystal connectivity matched against the chemical connectivity. Most trivial errors can be corrected by CCDC staff but, if necessary, assistance is requested of the author. If errors are still uncorrected when an entry is archived to the database then comments are introduced to alert users to the error status of the entry.

Some of the special arrangements between the CCDC and major journals will now be discussed.

## 3. Acta Crystallographica

Over the last few years structural papers published in sections B and C of Acta Crystallographica have been typeset from CIF files [1], prepared either by the Chester editorial staff or, increasingly, supplied directly by authors. For each issue of these sections the relevant CIF entries are transmitted to the CCDC where the information required for the database is extracted, converted to in-house format, and input to the standard validation software.

This arrangement has the obvious advantage that data are not re-keyboarded and the scheme has worked very well. There are, however, some problems associated with this form of data capture and these will be discussed later.

## 4. American Chemical Society

Policy regarding the publication of atomic coordinates by the American Chemical Society varies from journal to journal. However, if these data are not present in the printed paper, then they are available as Supplementary Material in microfiche. Discussions have been held with the editorial boards of certain ACS journals but, thus far, a special cooperation exists only between the CCDC and the Journal of Organic Chemistry.

For this journal, authors are required to submit an X-Ray Data Deposition form for each reported structure. When manuscripts have been accepted for publication, these forms are forwarded by JOC staff to the CCDC. However, “it is the author’s responsibility to supply the x-ray data itself to the CCDC after receiving notification of manuscript acceptance.” Such an arrangement inevitably creates supply problems and currently letters must be written to 85 % of authors requesting that they submit the data to the CCDC.

## 5. Royal Society of Chemistry

For many years, the Royal Society of Chemistry has forwarded to the CCDC all supplementary material which has been submitted with manuscripts accepted for publication. However, there has been growing pressure on certain editorial boards to discontinue the printing of tables of atomic coordinates. Discussions between the CCDC and various RSC journal managers have resulted in a new scheme to be implemented in 1996. The esssential features of this new regime are:

- The author submits to the journal the manuscript and data for deposition.
- The data for deposition can be submitted as: CCDC deposition form by e-mail (preferred option) or CCDC deposition form in hard copy together with the data.
- Data are provided to the referee as an electronic file, printout of an electronic file or hard copy.
- On acceptance for publication, the journal staff/editors will assign a CCDC deposition number which will be edited into the CCDC deposition form. This number will take the form, for example, 182/1234 where 182 is the CCDC coden for J. Chem. Soc., Chem. Commun.
- The following footnote will be printed in the published paper: “Crystallographic data (excluding structure factors) for the structure(s) reported in this paper have been deposited with the Cambridge Crystallographic Data Centre as supplementary publication number 182/1234. Copies of the data can be obtained, free of charge, on application to the Director.…”

It is hoped that this new arrangement will stimulate an increase in data deposition by electronic means.

## 6. CCDC Deposition Form

Copies of the form mentioned in the above section can be obtained in two ways:

- Send e-mail to fileserv@chemcrys.cam.ac.uk with the message sendme depform
- On World Wide Web connect to the CCDC Home Page http://www.ccdc.cam.ac.uk and download the form.

## 7. Private Communications

A number of users of the database have indicated to us that they possess many data sets corresponding to crystal structures which they do not intend to publish but which they would like to deposit in the database. This situation may well increase in the future as a result of fast data collection techniques coupled with more powerful structure-solving software.

We welcome this offer of data and have announced that data sets can be e-mailed to the address deposit@chemcrys.cam.ac.uk. The original submissions will be archived unchanged and data relevant to the database extracted and input to the standard validation software. When archived to the database, each entry will be labeled as a private communication, indicating to users that it has not been subject to journal refereeing. At the request of an industrial user, it has been agreed to include a statement of the type “contributed by the ABC Company, New Town, New Jersey, USA.”

## 8. Problems and Challenges

Although we are keen to promote the deposition of data by electronic means, we are under no illusions that handling such data will be trouble-free. Our experience to date indicates frequent problems associated with the completion of forms, file formats and the data content of CIF files.

The CCDC data deposition form was, in our opinion, carefully constructed and examples were provided on how to complete each section. In spite of succinct instructions, we have been surprised by the great variety of ways in which users have completed the form. While software can be designed to cater for many of these variants, undoubtedly visual checking will also be necessary.

Most atomic coordinates are submitted either in CIF or SHELX formats and the conversion of these to CCDC format is relatively trouble-free. However, we continue to receive many data sets in “plain text” akin to tables in a printed paper, complete with captions, titles etc. It seems likely that most of these will require manual editing rather than interpretation by a computer program.

The principal difficulties associated with CIF files concern those data which are entered manually by the author as opposed to the data generated from the structure solution and refinement packages. Although the International Union of Crystallography has made publically available the CIF Dictionary, nevertheless data values are often associated with incorrect data names, units such as Kelvin and Celsius are incorrectly used and syntax errors are quite frequent. Until the user community has gained more experience in the construction of CIF files, we cannot expect software packages to handle these correctly in a purely automatic fashion.

In spite of these numerous problems, we feel confident that the advantages for database building afforded by electronic deposition far outweigh the difficulties to be overcome.
